# An epigenetic auto-feedback loop regulates TGF-β type II receptor expression and function in NSCLC

**DOI:** 10.18632/oncotarget.4893

**Published:** 2015-08-12

**Authors:** Shanzhong Yang, Yong-Jig Cho, Lin Jin, Guandou Yuan, Arunima Datta, Phillip Buckhaults, Pran K. Datta

**Affiliations:** ^1^ Division of Hematology and Oncology, Department of Medicine, Comprehensive Cancer Center, University of Alabama at Birmingham, Birmingham, AL, USA; ^2^ Birmingham Veterans Affairs Medical Center, Birmingham, AL, USA; ^3^ Department of Surgery, Vanderbilt University School of Medicine, Nashville, TN, USA

**Keywords:** c-Myc, miR-20a, TGF-β, TGF-β type II receptor, miR-145

## Abstract

The downregulation of transforming growth factor-β (TGF-β) type II receptor (TβRII) expression and function plays a pivotal role in the loss of the TGF-β-induced tumor suppressor function that contributes to lung cancer progression. The aberrant expression of miRNAs has been shown to be involved in the regulation of oncogenes and tumor suppressor genes. Our current study involving miRNA microarray, northern blot and QRT-PCR analysis shows an inverse correlation between miR-20a and TβRII expression in non-small cell lung cancer (NSCLC) tissues and cell lines. Stable expression of miR-20a downregulates TβRII in lung epithelial cells which results in an inhibition of TGF-β signaling and attenuation of TGF-β-induced cell growth suppression and apoptosis. Stable knock down of miR-20a increases TβRII expression and inhibits tumorigenicity of lung cancer cells *in vivo*. Oncogene c-Myc promotes miR-20a expression by activating its promoter leading to downregulation of TβRII expression and TGF-Δ signaling. MiR-145, which is upregulated by TGF-β, inhibits miR-20a expression by targeting c-Myc and upregulates TβRII expression. These correlations among miRNAs and cellular proteins are supported by TCGA public database using NSCLC specimens. These results suggest a novel mechanism for the loss of TβRII expression and TGF-β-induced tumor suppressor functions in lung cancer through a complex auto-feedback loop TGF-β/miR-145/c-Myc/miR-20a/TβRII.

## INTRODUCTION

Transforming growth factor-β (TGF-β) belongs to a superfamily of structurally related polypeptides that are involved in various biological processes, including cell growth, differentiation, angiogenesis, apoptosis, and extracellular matrix remodeling. The multifunctional effects of TGF-β in cellular actions occur by binding to its receptors with intrinsic serine/threonine kinase activity. TGF-β initiates signals by binding to TGF-β receptor II (TβRII) and stabilizes the heteromeric complex with TGF-β receptor I (TβRI). TβRI, thus being activated, propagates the signals to intracellular signal mediators including Smads [1–3]. TGF-β-mediated tumor suppressor functions are frequently lost in lung cancer, which may play a pivotal role in the pathogenesis of lung cancer. The unresponsiveness of lung cancer cells to TGF-β could be caused by loss of TβRII function [4, 5]. Mutations or deletion within the coding sequence of the TβRII gene are rare in non-small cell lung cancer (NSCLC). Mutations in Smad2 and Smad4 genes have been found in less than 10% of lung cancers [6, 7]. However, It has been shown that TβRII expression is decreased or lost in 80% of squamous cell carcinoma, 42% adenocarcinoma, and 71% large cell carcinoma [8]. Gene expression profiles are dependent on changes in the epigenome, including DNA methylation, histone modifications, and noncoding RNA regulation. Many studies have shown that inhibition of DNA methylation and histone deacetylation can increase some tumor suppressor gene expression in lung cancer [9, 10]. Our previous study shows that there is a high level of chromatin deacetylation in the promoter of TβRII gene and histone deacetylase inhibitors can in part restore TβRII expression and TGF-β-induced tumor suppressor functions in lung cancer cells [11]. These findings suggest that cancer cells could escape from the autocrine growth inhibitory effect of TGF-β due to loss of expression of TβRII through epigenetic deregulation.

Recent studies have shown that non-coding RNAs (ncRNAs) participate in epigenetic regulation of genes and play a very important role in developmental and tissue specific gene expression. Aberrant regulation of ncRNAs can lead to developmental abnormalities and a variety of diseases including cancer [12–14]. NcRNAs are loosely grouped into two major classes based on transcript size: small and long ncRNAs. Small ncRNAs are mainly represented by the well-documented miRNAs. MiRNAs are ~22 nucleotides (nt) long and mainly regulate protein expression of specific mRNA by either translational inhibition or transcript degradation [15]. MiRNAs are frequently aberrantly expressed in lung carcinoma tissues, indicating that they are involved in development and progression of lung cancer [16–19]. Several miRNAs have been reported to be upregulated (like miR-21, miR-17–92 and miR-221/222) or downregulated (like miR-34a-c, miR-29, let-7/miR-98, miR-15/16, miR128b, miR-200/429, miR-197, miR-93 and miR-126) in lung cancer [18]. These miRNAs regulate a number of cellular processes through targeting different genes including differentiation, cell proliferation, apoptosis, migration, and invasion that contribute to tumor development and progression. Although significant progress has been made in this new field of research, much about miRNAs, their targets and biological functions are unknown.

Recent studies have shown that ncRNAs play a very important role in epigenetic regulation of tumor suppressor genes and oncogenes. Therefore, increased attention is now being placed to study the role of these ncRNAs in the initiation and progression of human cancers. As described above, histone deacetylase inhibitors can, only in part, restore TβRII expression in lung cancer cells [11], indicating that other mechanisms may be involved in the downregulation of TβRII in lung cancer. To get a complete picture of the mechanism of downregulation of TβRII in lung cancer, we performed qRT-PCR-based miRNA microarray analyses using lung cell lines with different levels of TβRII expression. We found that miRNA-20a, which belongs to the polycistronic cluster miR-17–92 at 13q31.3, overexpresses in lung cancer cells lacking TβRII. According to the miRNA target database, TβRII is a miR-20a target. MiR-20a has also been shown to be upregulated in lung cancers [20]. So we hypothesize that an increased level of miR-20a may contribute to TβRII downregulation in lung cancer. Our current study shows that miR-20a down-regulates TGF-beta receptor II expression and modulates TGF-beta signaling in lung cancer cells. Oncogenic c-Myc inhibits TβRII expression by upregulating miR-20a. MiR-145, a TGF-β signaling target, decreases miR-20a expression and upregulates TβRII through c-Myc. Our findings indicate that interaction between TGF-β/miR-145 and Myc/miR-20a plays a role in NSCLC progression through downregulation of TβRII.

## RESULTS

### TβRII and miR-20a expressions are inversely correlated in lung cancer

**Figure 1:**
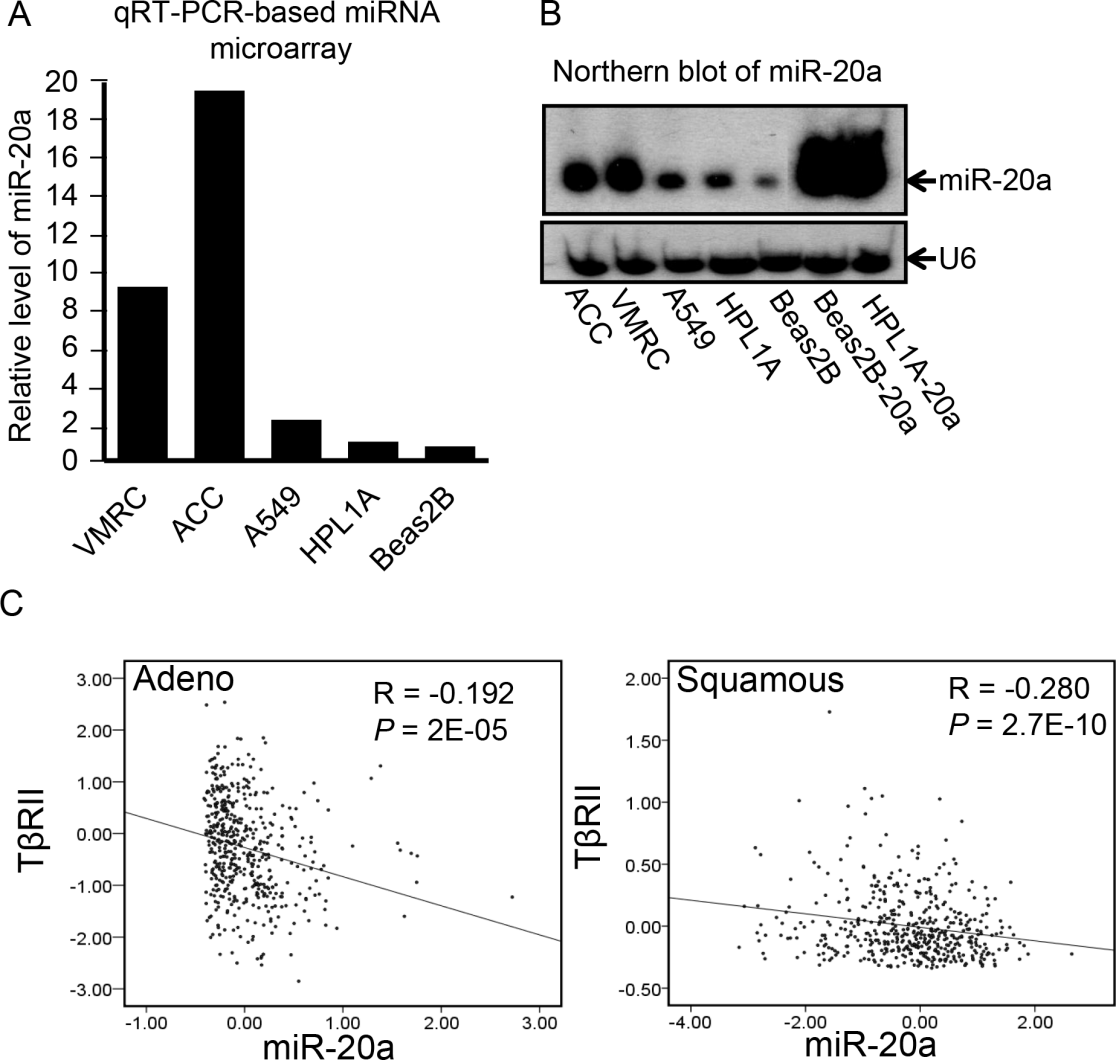
The expression of TβRII and miR-20a are inversely correlated in lung cancer **A.** Total RNA was purified from human lung cancer cell lines A549, ACC-LC-176 (ACC) and VMRC-LCD (VMRC) and immortalized normal lung epithelial cell lines Beas2B and HPL1A. Expression of miRNAs was analyzed by microarray. **B.** MiR-20a levels in human lung cell lines A549, ACC, VMRC, Beas2B and HPL1A, and Beas2B and HPL1A cells with miR-20a stable expression (Beas2B-20a and HPL1A-20a as positive control) were analyzed by northern blotting. Small non-coding RNA U6 was used as loading control. **C.** RNA-seq based exon expression data for TΔRII and miR-20A were obtained from 488 lung adenocarcinoma patients and 490 lung squamous carcinoma patients and analyzed for association by Spearman-rank. A *P*-value of *P* < 0.05 was considered significant. The correlation coefficients (R) and associated *P*-values are shown. The scatter plot demonstrates the correlation between two continuous variables.

To explore whether miRNA dysregulation in lung cancer is involved in TβRII downregulation, we performed miRNA microarray analyses using total RNA from lung adenocarcinoma cell line A549, immortalized human lung epithelial cell lines Beas2B and HPL1A with normal expression of TβRII, lung squamous carcinoma cell line ACC-LC-176 (ACC), and adenocarcinoma cell line VMRC-LCD (VMRC) lacking TβRII expression. As shown in Fig. 1A, there are higher levels of miR-20a expression in ACC and VMRC cells compared with that in A549, Beas2B, and HPL1A cells. To verify the microarray data, we performed northern blot analyses using 20 μg total RNA from each cell line, which confirms the result (Fig. 1B). These data indicate that there is an inverse correlation between TβRII and miR-20a expression in lung cells. To further investigate whether this correlation exists in lung tumor tissues, we have performed in-depth analyses of the publicly available The Cancer Genome Atlas (TCGA) gene expression (RNA-seq) data base (http://genome-cancer.ucsc.edu/). For these analyses, we have used the expression data for TβRII and miR-20a from a total of 488 primary lung adenocarcinoma specimens and 490 primary lung squamous carcinoma specimens. As shown in Fig. 1C, there is a significant inverse correlation between the expression of TβRII and miR-20a in both adenocarcinoma and squamous cell carcinoma. These results suggest that high level of miR-20a may be responsible for TβRII downregulation in NSCLC.

### MiR-20a downregulates TβRII expression in lung cell lines through binding to its 3′-UTR

**Figure 2:**
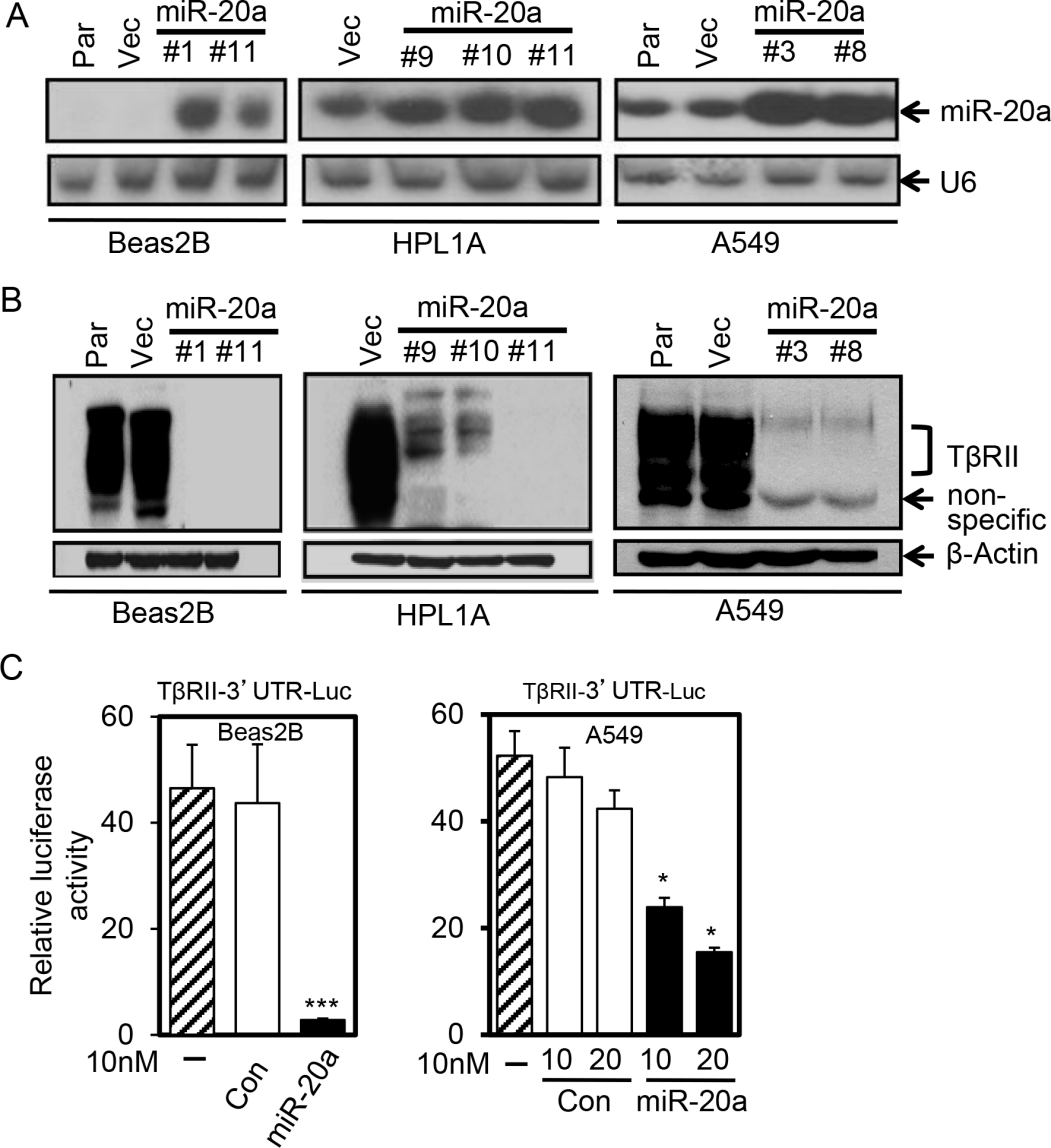
MiR-20a inhibits TβRII expression in lung cells by binding to its 3′-UTR **A.** Total RNA from A549, Beas2B and HPL1A cells stably expressing miR-20a and their parental (Par) and control vector cells (Vec) was analyzed for miR-20a expression by northern blotting. **B.** Cell lysates from the cells in “Figure 2A” was analyzed for TβRII protein expression by western blotting. **C.** Beas2B and A549 cells were co-transfected with miRNA reporter vector containing the sequence of TβRII gene 3′-UTR with miR-20a binding site and miR-20a mimics or control mimics. Normalized luciferase activities were plotted. *P < 0.05, ***P < 0.001, *vs*. control group (Con).

To explore whether miR-20a overexpression can downregulate the expression of TβRII in lung cells, we established A549, Beas2B, and HPL1A cell clones stably expressing miR-20a using lenti-virus. As shown in Fig. 2A, there are strong expressions of miR-20a in A549, Beas2B, and HPL1A cell clones compared to their parental and control vector cell clones. The above cell clones with miR-20a stable expression show decreased levels of TβRII protein in western blot analyses (Fig. 2B), suggesting that miR-20a downregulates TβRII expression. In order to test whether miR-20a regulates TβRII expression by directly binding to TβRII mRNA 3′-UTR, we constructed miRNA reporter vector by inserting TβRII mRNA 3′ untranslated region (3′-UTR) (from 37 to 2129) containing a miR-20a binding site into downstream of luciferase gene of pGL3 control vector. We co-transfected the reporter vector with miR-20a mimics or control miRNA mimics into A549 and Beas2B cells. 48 h after transfection, we harvested the cells, measured luciferase activity and β-Gal activity, and normalized luciferase activity to β-Gal activity. As shown in Fig. 2C, miR-20a suppressed luciferase activity in both cell lines when compared with that in cells transfected with control miRNA mimics. These results suggest that miR-20a downregulates TβRII by directly binding to 3′-UTR.

### Stable expression of miR-20a attenuates TGF-β/Smad signaling

**Figure 3:**
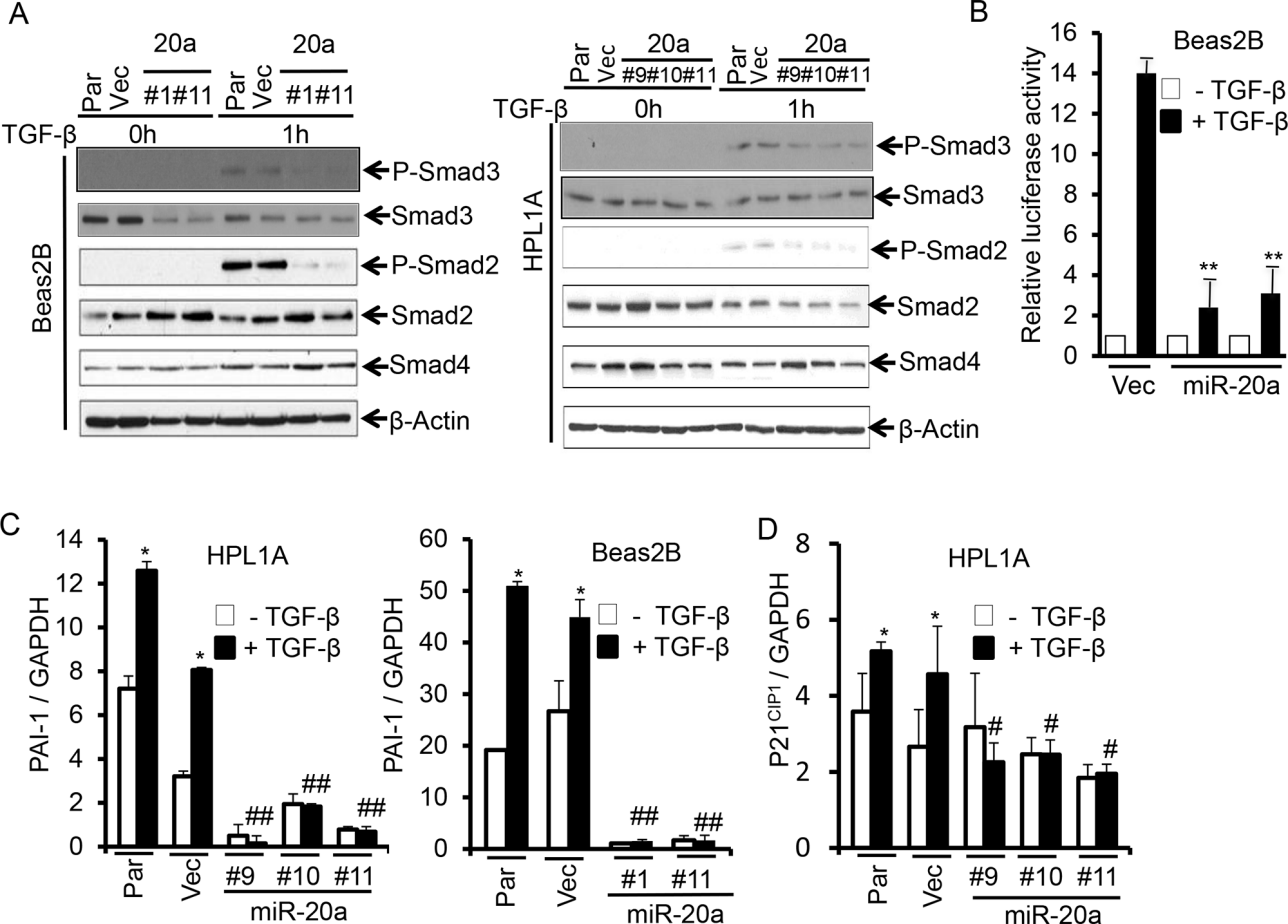
MiR-20a attenuates TGF-β/Smad signaling **A.** Beas2B and HPL1A cell clones with miR-20a stable expression and their parental and control vector cells were serum starved for 2 h and then treated with 5 ng/ml TGF-β for 1 h. Indicated proteins were analyzed by western blotting. **B.** Beas2B cells with miR-20a stable expression and control vector cells were transfected with TGF-β responsive luciferase vector (CAGA)_9_ MLP-Luc. Transfeted cells were treated with 5 ng/ml TGF-β for 22 h. Luciferase and Δ-galactosidase activities were measured. **P < 0.01, *vs*. TGF-β treated vector cells (Vec). **C.** and **D.** QRT-PCR analysis was performed with total RNA from cells in “Figure 3A” treated with 5 ng/ml TGF-β for 5 h. *P < 0.05, *vs*. TGF-β untreated cells in each group, #*P* < 0.05, ##*P* < 0.01, *vs*. TGF-β treated cells in parental and vector groups.

We have observed that HPL1A and Beas2B cells express low levels of miR-20a and appreciable levels of TβRII and are responsive to TGF-β. To test whether miR-20a has any effect on TGF-β/Smad signaling, we first analyzed the levels of phosphorylation of Smad2 and Smad3 in Beas2B and HPL1A lung epithelial cells treated with TGF-β. As shown in Fig. 3A, the induction in phosphorylation of Smad2 and Smad3 is diminished in cells with miR-20a stable expression compared with that in control cells. To investigate the effect of miR-20a on TGF-β/Smad-mediated transcriptional activity, we transfected luciferase reporter vector with TGF-β response element [(CAGA)_9_ MLP-Luc] into Beas2B cells stably expressing miR-20a and control vector cells. After cells were exposed to TGF-β, luciferase activity in control vector cells is significantly higher (14 fold) than that in cells with miR-20a overexpression (~2 fold) (Fig. 3B). To further investigate the effect of miR-20a on TGF-β inducible target genes, we treated the above stable cell clones with miR-20a overexpression or control vector with TGF-β and then analyzed the expressions of mRNA of p21^CIP1^ and PAI-1. As shown in Fig. 3C and 3D, TGF-β promotes expressions of PAI-1 and p21^CIP1^ mRNA in parental and control vector cells. However, there is no difference in the levels of PAI-1 and p21^CIP1^ mRNAs in cell clones expressing miR-20a with and without TGF-β treatment. To investigate whether miR-20a directly regulates TGF-β signaling through downregulating TβRII expression, we transfected TβRII expression vector to Beas2B cells with miR-20a overexpression. As shown in Figure S1A and S1B, TβRII re-expression attenuates inhibitory effect of miR-20a on p21^CIP1^ and PAI-1 expression. These results indicate that miR-20a can directly inhibit TGF-β signaling by downregulating TβRII expression.

### MiR-20a inhibits TGF-β induced cell growth inhibition and apoptosis

**Figure 4:**
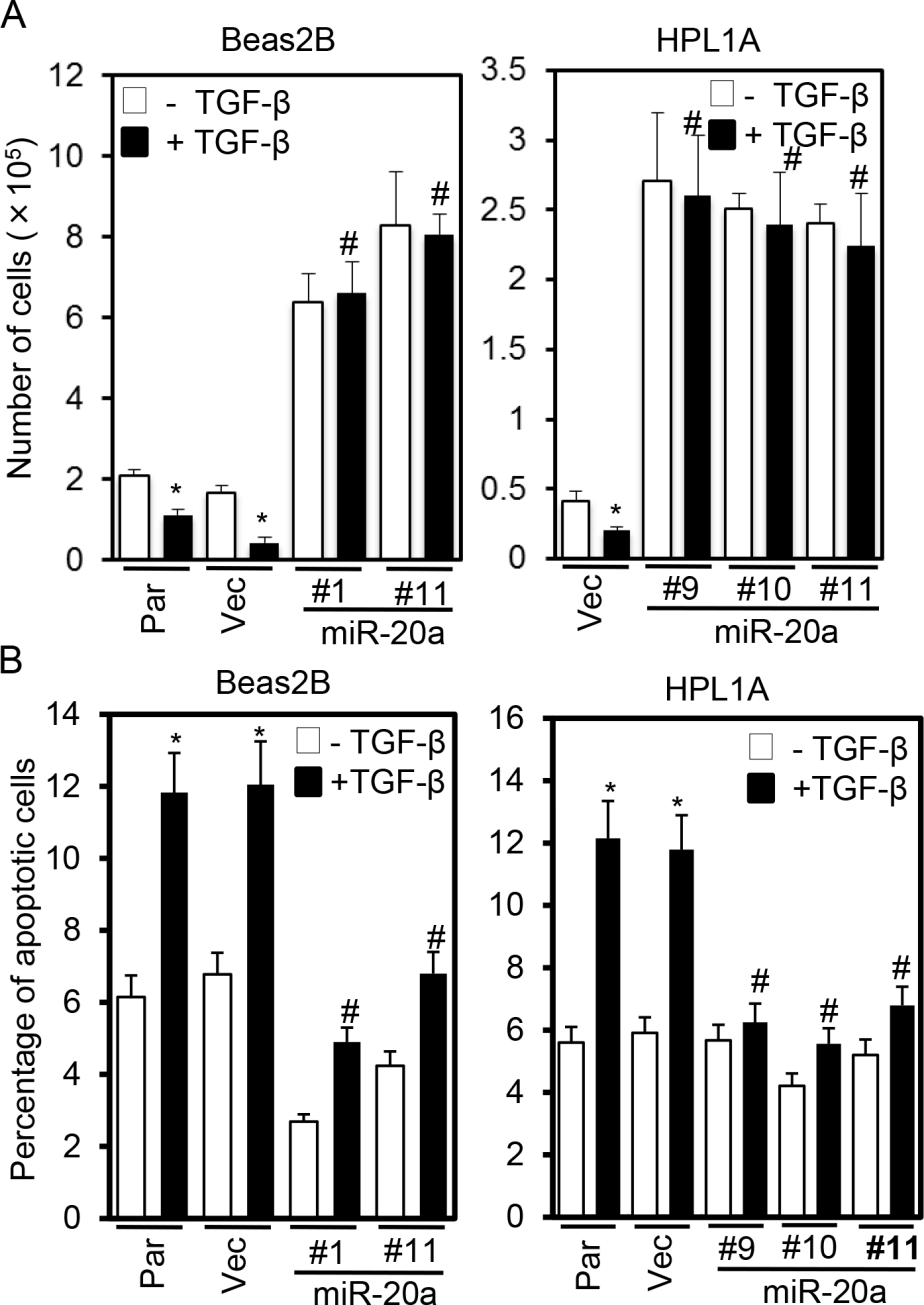
Stable expression of miR-20a inhibits TGF-β-induced growth inhibition and apoptosis **A.** Beas2B and HPL1A cells stably expressing miR-20a and parental and control vector cells were treated with 5 ng/ml TGF-β for 5 days. Cell numbers from 5^th^ day count were plotted. *P < 0.05, *vs*. TGF-β untreated cells; #*P* < 0.05, vs. TGF-β treated cells in parental and vector groups. **B.** Cells in “Figure 4A” were treated with 5 ng/ml TGF-β for 40 h and then harvested. Apoptotic cells were analyzed by FACS. *P < 0.05, *vs*. TGF-β untreated cells; #*P* < 0.05, *vs*. TGF-β treated cells in Par and Vec groups.

TGF-β signaling can inhibit cell growth and induce apoptosis by regulating several target gene expressions [3, 21]. To test whether tumor suppressor functions of TGF-β are inhibited by miR-20a, we first performed growth assays by counting cells. Beas2B and HPL1A parental cells, vector control cells, and miR-20a overexpressing clones were treated with 5 ng/ml TGF-β for five days, and then the cell numbers were counted every day. MiR-20a expression strongly induces growth of Beas2B and HPL1A cells (Fig. 4A, data from 5^th^ day count). TGF-β inhibits growth of parental and vector control cells whereas it has no effect on miR-20a overexpressing clones (Fig. 4A). Interestingly, the basal level of growth of miR-20a expressing clones is much higher than that of control cells suggesting that the endogenous TGF-β may play a role in inhibiting the growth. To determine whether miR-20a has any effect on TGF-β-mediated apoptosis, we performed FACS analyses after 5 ng/ml TGF-β treatment for 40 h. As shown in Fig. 4B, TGF-β significantly induces apoptosis in vector control and parental Beas2B and HPL1A cells, whereas miR-20a expression attenuates TGF-β-induced apoptosis. These results suggest that miR-20a reduces tumor suppressor functions of TGF-β by downregulating TβRII.

### Knockdown of miR-20a in lung cancer cells inhibits tumorigenicity by restoring TβRII expression

**Figure 5:**
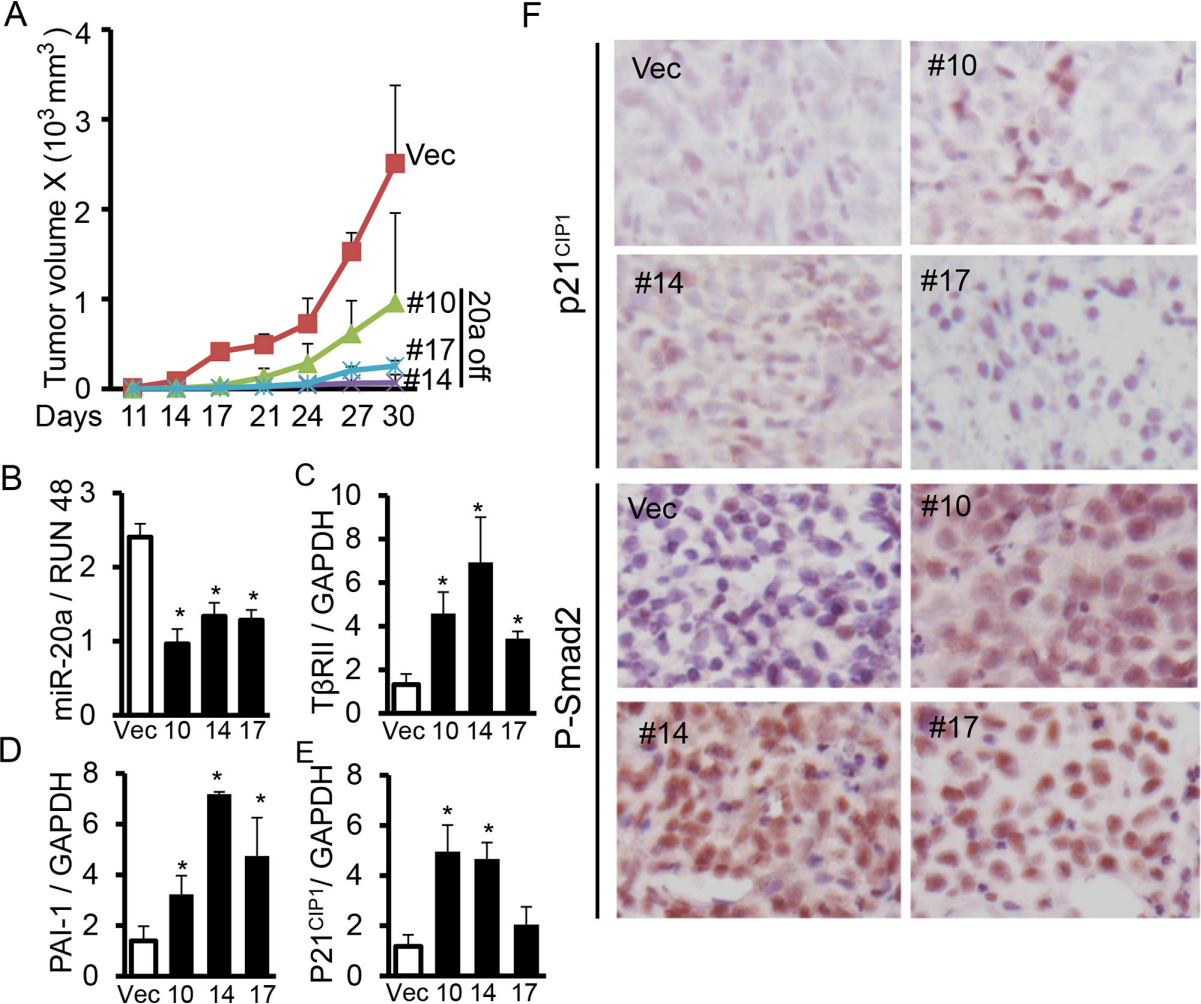
Knocking down miR-20a expression upregulates TβRII and inhibits tumorigenecity *in vivo* **A.** Human lung cancer cells ACC-LC-176 with miR-20a knockdown and control vector cells were subcantaneously injected into nude mice. Tumor length (L) and width (W) were measured every three days. Tumor volumes were calculated using the equation *V* = 0.5LW^2^. Growth curves for tumors were plotted as the mean volume ± SD of tumors of mice from each group. **B, C, D.** and **E.** Expression of miR-20a, TβRII, p21^CIP1^ and PAI-1 in tumor xenografts were analyzed by QRT-PCR. *P < 0.05, *vs*. vector group. **F.** Immunohistochemical analyses for the expression of p21^CIP1^ and P-Smad2 in xenograft tumors were performed. Representative pictures are shown (X 400).

Since there are higher levels of miR-20a in lung cancer cell lines ACC-LC-176 and VMRC-LCD lacking TβRII expression (Fig. 1A and 1B), we hypothesize that miR-20a downregulation in these cells can decrease tumorigenicity by restoring TβRII expression and TGF-β-mediated tumor suppressor functions. We established stable miR-20a knockdown ACC-LC-176 cell lines and subcutaneously injected them into nude mice. We monitored tumor growth *in vivo*. As shown in Fig. 5A, miR-20a knockdown decreases the tumor volume significantly compared to the control vector. Cell proliferation marker Ki67 is decreased and apoptotic marker cleaved caspase 3 is increased in xenograft tumor tissues resulting from miR-20a knock down (Fig. S2). QRT-PCR analyses with tumors show that inhibition of miR-20a expression, in part, restores TβRII expression (Fig. 5B and 5C). This is associated with an increase in the levels of PAI-1 and p21^CIP1^ expression in the tumors derived from knockdown cells (Fig. 5D, and 5F upper panels). The increase in the endogenous TGF-β signaling in tumors is evident from the nuclear localization of Smad2 (Fig. 5F lower panels). Similarly, stable knockdown of miR-20a in VMRC-LCD cells increased TΔRII expression (Fig. S3). To test the specificity of whether these effects of miR-20a are through TβRII/TGF-β signaling, we performed anchorage-independent growth assays using these cell lines with stable knockdown of miR-20a after treated with 10 μM TGF-Δ receptor kinase inhibitor LY2109761 (LY). As shown in Fig. S4, the number of clones in cells with miR-20a downregulation is lower than that in parental and control vector cells, suggesting that endogenous TGF-β signaling is established due to restoration of TΔRII. LY treatment does not affect colony formation in parental and control vector cells, whereas miR-20a knockdown clones showed significant increase in colony formation due to blockade of TGF-β signaling. These results suggest that miR-20a upregulation in lung cancer cells increases tumorigenicity through downregulation of TΔRII and loss of TGF-β tumor suppressor functions.

### C-Myc downregulates TβRII by inducing the expression of miR-20a

**Figure 6:**
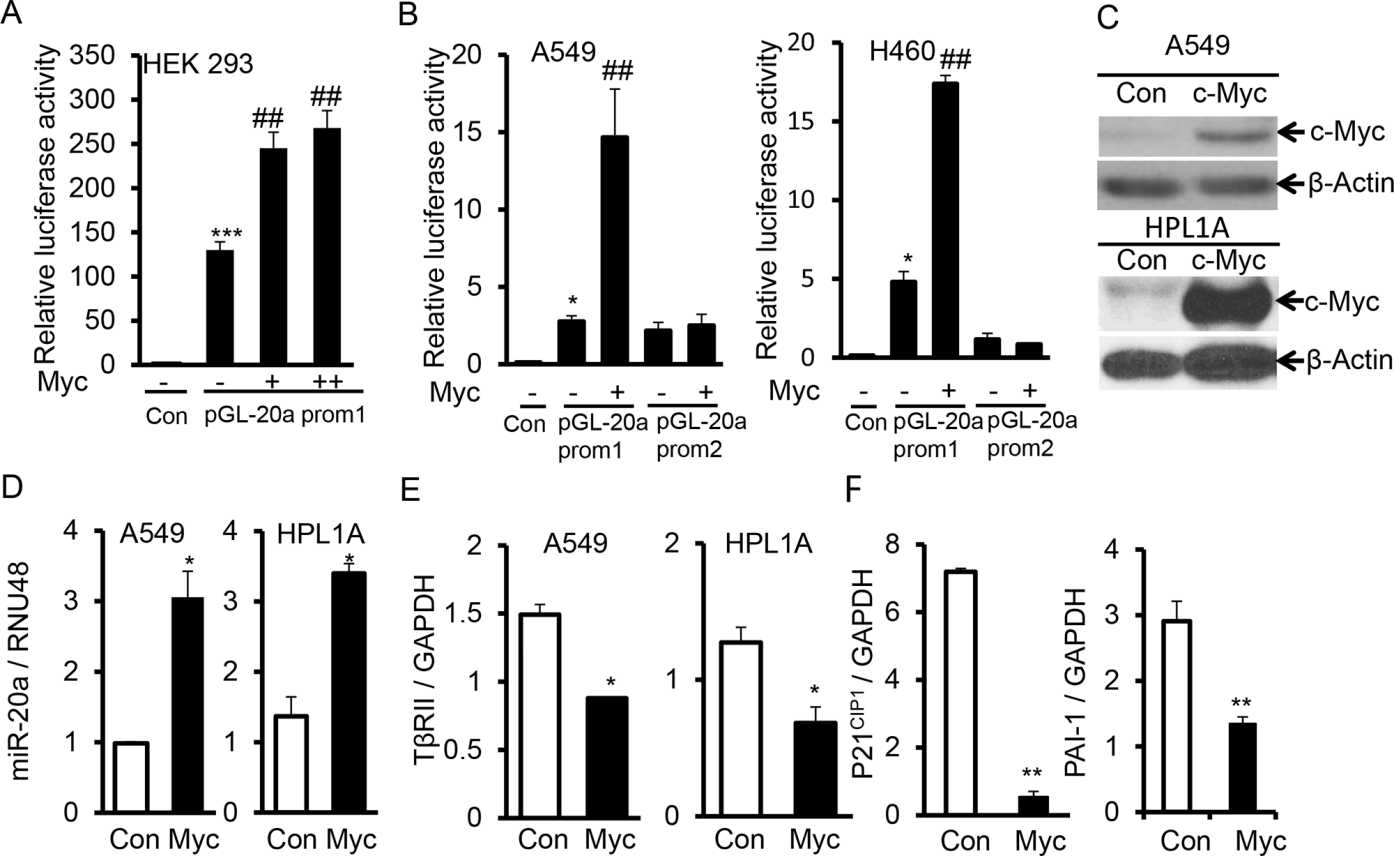
C-Myc downregulates TβRII and inhibits TGF-β signaling through inducing miR-20a expression **A.** PGL luciferase reporter vector with miR-20a promoter containing c-Myc binding site (pGL-20a prom1) and different concentrations of c-Myc expression vector were co-transfected into HEK-293 cells. Normalized luciferase activities were plotted. ***P < 0.001, *vs*. control cells; ##*P* < 0.01, *vs*. cells transfected only with pGL-20a prom1. **B.** PGL luciferase reporter vector, pGL-20a prom1 or without c-Myc binding site (pGL-20a prom2) and c-Myc expression vector were co-transfected into A549 and H460 cells. Normalized luciferase activities were plotted. *P < 0.05, *vs*. control cells; ##*P* < 0.01, *vs*. cells transfected only with pGL-20a prom1. **C.** A549 and HPL1A cells were transfected with c-Myc expression vector. Two days after transfection, c-Myc expression levels were analyzed by western blotting. **D, E.** and **F.** Total RNA was purified from cells transfected as described in “Fig. 6C” and QRT-PCR was performed. *P < 0.05, **P < 0.01, *vs*. control cells.

Previous study shows that c-Myc can promote the expression of miR-17–92 family in transcriptional level [22]. There is a c-Myc binding site in the upstream of miR-17–92 gene promoter. To verify whether c-Myc can induce miR-20a transcriptionally, we constructed luciferase reporter vectors containing the 5′ end promoter sequence of miR-17–92 gene with or without c-Myc binding site. We co-transfected them with c-Myc expression vector into HEK-293, A549, and H460 cell lines. As shown in Fig. 6A, c-Myc overexpression increases the promoter (prom 1, with c-Myc binding site) activity of miR-17–92 gene. However c-Myc doesn't affect the activity of the miR-17–92 gene promoter without c-Myc binding site (prom 2), indicating that c-Myc directly regulates the promoter activity of miR-17–92 through binding to its promoter (Fig. 6B). To investigate whether there is a correlation between c-Myc and miR-20a expression in lung cancer cells, we analyzed the levels of c-Myc expression in A549, Beas2B, HPL1A, ACC-LC-176, and VMRC-LCD cells with different levels of miR-20a by QRT-PCR analyses (Figure S5A). Interestingly, ACC-LC-176 and VMRC-LCD cells with high levels of miR-20a show increased c-Myc mRNA expression, compared to A549, Beas2B, and HPL1A cells with low levels of miR-20a (Fig. S5A). To test whether c-Myc can inhibit TβRII expression through upregulating miR-20a, we transfected c-Myc expressing vector to A549 and HPL1A cells. As shown in Fig. 6C, there are higher levels of c-Myc protein in A549 and HPL1A cells transfected by the c-Myc expression vector, compared to control vector cells. C-Myc overexpression in A549 and HPL1A cells promotes miR-20a expression and decreases the levels of TβRII mRNA in A549 and HPL1A cells (Fig. 6D and 6E). Downregulation of TΔRII by c-Myc resulted in the reduced expression of TGF-β target genes, p21^CIP1^ and PAI-1 (Fig. 6F). To further investigate the specific role of c-Myc in the upregulation of miR-20a in lung cancer cells, we knocked down c-Myc in ACC cells with high levels of miR-20a and c-Myc using c-Myc siRNA. As shown in Figure S5B, c-Myc protein expression in ACC cells is inhibited by c-Myc siRNA. C-Myc downregulation decreases miR-20a expression and promotes TβRII expression in ACC cells (Fig. S5C). To investigate whether c-Myc directly regulates TGF-β signaling through inhibiting TβRII expression, we co-transfected c-Myc expression vector with or without TβRII expression vector to HPL1A cells. As shown in Fig. S6A and S6B, TβRII re-expression decreases inhibitory effect of c-Myc on PAI-1 and p21^CIP1^ expression. These results suggest that oncogenic c-Myc attenuates TGF-β mediated tumor suppressor function through upregulating miR-20a expression and decreasing TβRII expression.

### MiR-145 downregulates miR-20a expression and restores TGF-β signaling by targeting c-Myc

**Figure 7:**
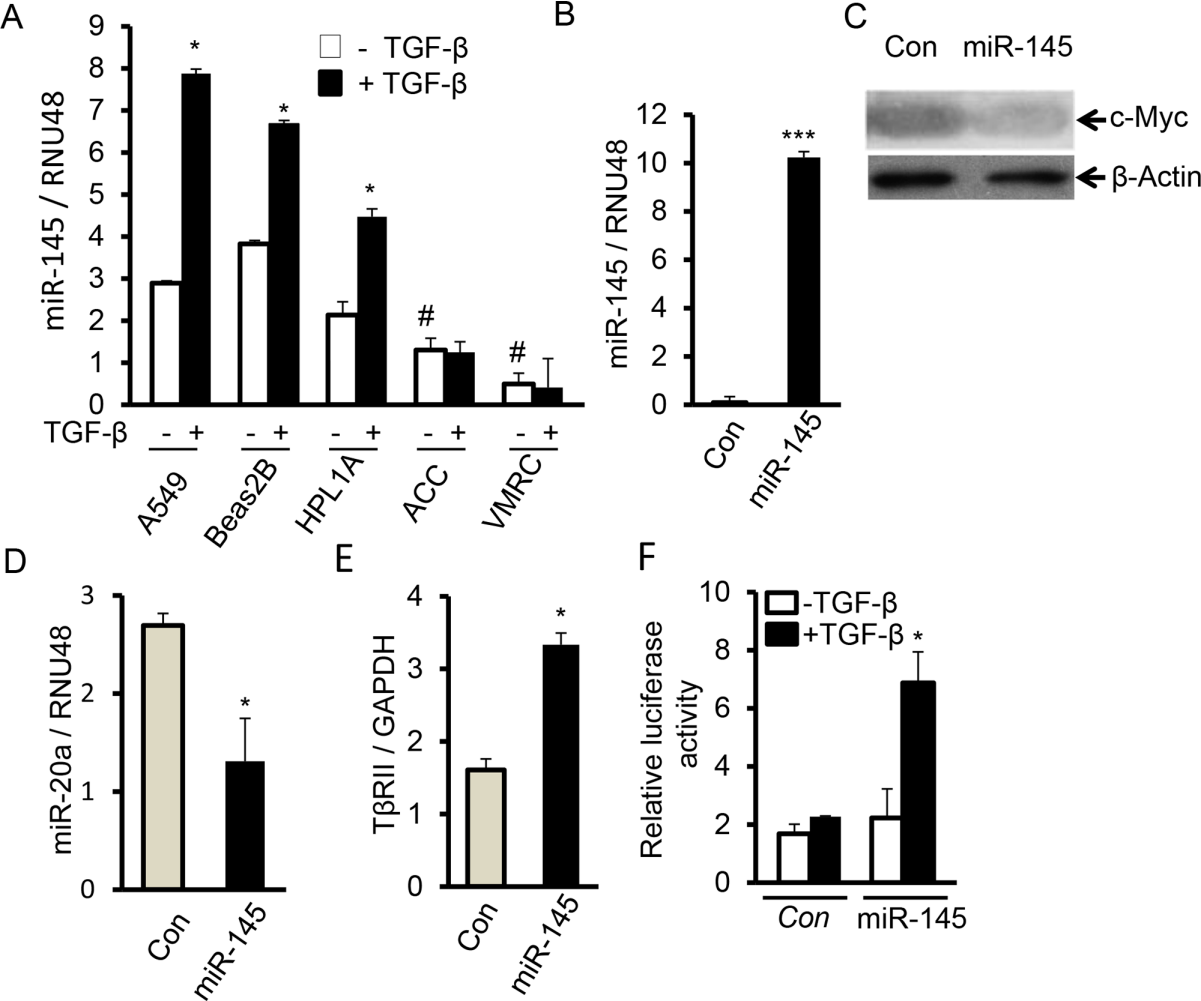
MiR-145 downregulates miR-20a and induces TΔRII by targeting c-Myc **A.** A549, Beas2B, HPL1A, ACC-LC-176 and VMRC-LCD cells were serum starved for 2 h and then treated with 2 ng/ml TGF-β for 24 h. Expression of miR-145 was analyzed by QRT-PCR. *P < 0.05, *vs*. TGF-β untreated cells; #*P* < 0.05, *vs*. TGF-β untreated A549, Beas2 and HPL1A cells. **B.** Total RNA was purified from VMRC cells stably expressing miR-145 and control vector cells. Expression of miR-145 was analyzed by QRT-PCR. ***P < 0.001, *vs*. control cells. **C.** C-Myc protein levels in Cell lysates from VMRC cells stably expressing miR-145 and control vector cells were analyzed by western blotting. **D** and **E.** MiR-20a and TβRII levels in VMRC cells stably expressing miR-145 (from Figure 7B) and control cells were measured by QRT-PCR. *P < 0.05, *vs*. control cells. **F.** VMRC cells stably expressing miR-145 and control cells were transfected with TGF-β responsive vector (CAGA)_9_ MLP-Luc and cells were treated with 5 ng/ml TGF-β for 24 h. Normalized luciferase activities were plotted. *P < 0.05, *vs*. TGF-β untreated cells in each group.

Previous studies have shown that TGF-β signaling promotes miR-145 expression [23, 24], and miR-145 directly targets c-Myc [25, 26]. There is low expression of miR-145 in lung cancer [27, 28]. We hypothesized that a low level of miR-145 may contribute to high levels of c-Myc and miR-20a in lung cancer. To test this hypothesis, we first analyzed miR-145 expression in lung cancer cells with different levels of miR-20a and c-Myc expression. Indeed, as shown in Fig. 7A, there are higher levels of miR-145 in A549, Beas2B, and HPL1A cells with low levels of c-Myc and miR-20a, compared with those in ACC and VMRC cells with high levels of c-Myc and miR-20a. TGF-β promotes miR-145 expression in A549, Beas2B and HPL1A cells with normal expression of TβRII. When miR-145 was expressed into VMRC and ACC cells, expression of c-Myc and miR-20a was inhibited and TβRII expression was upregulated (Fig. 7 and Fig. S7). To investigate whether miR-145 can promote TGF-β signaling by downregulating c-Myc and upregulating TβRII, we transfected a TGF-β signaling reporter vector into VMRC cells stably expressing miR-145 and control vector cells. As shown in Fig. 7F, luciferase activity in VMRC cells with miR-145 overexpression is significantly higher than that in control vector cells after cells were treated with TGF-β for 24 h. The results suggest that miR-145 can promote TGF-β signaling by downregulating c-Myc and miR-20a and restoring TβRII expression.

### There are correlations in expressions of these proteins and miRNAs in lung tumors

**Figure 8:**
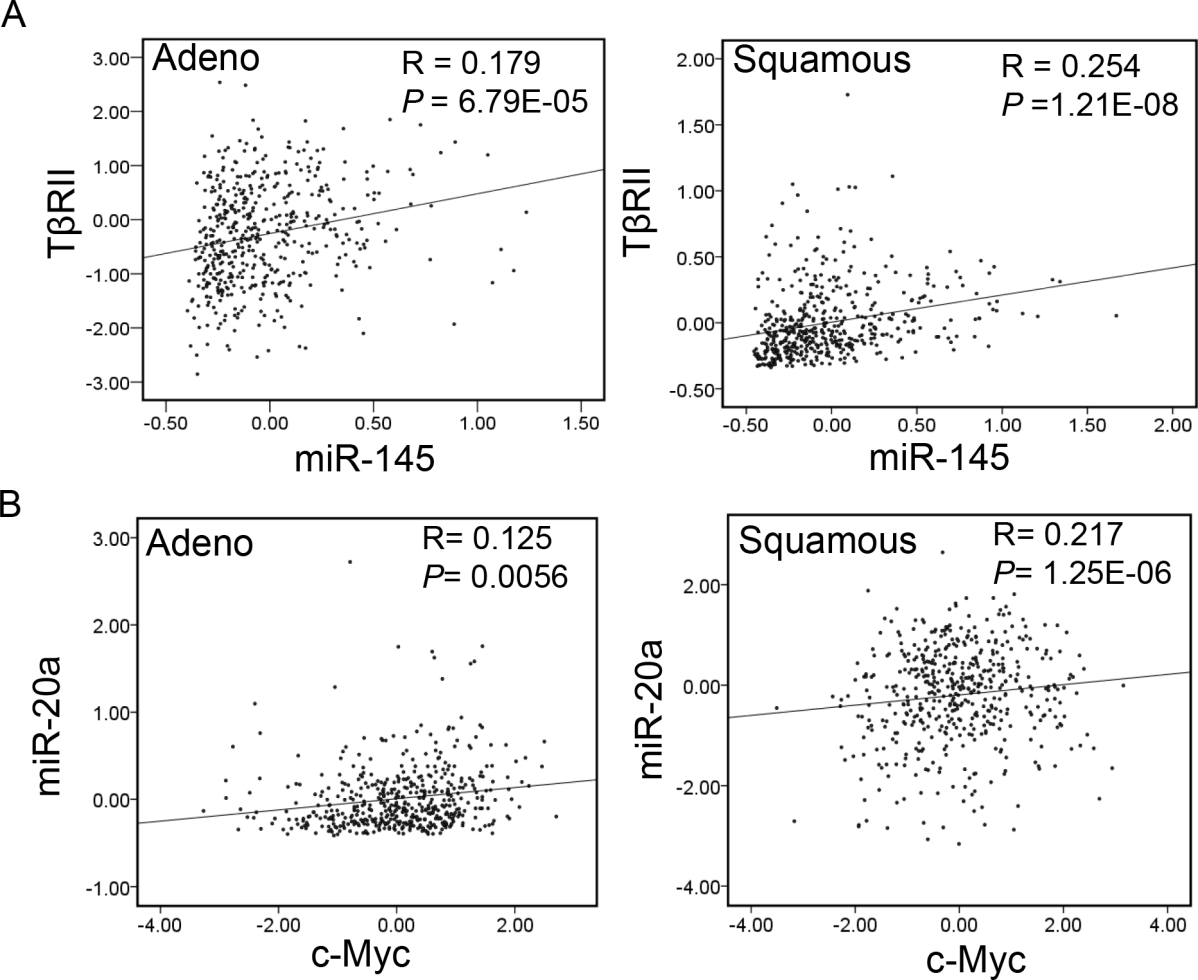

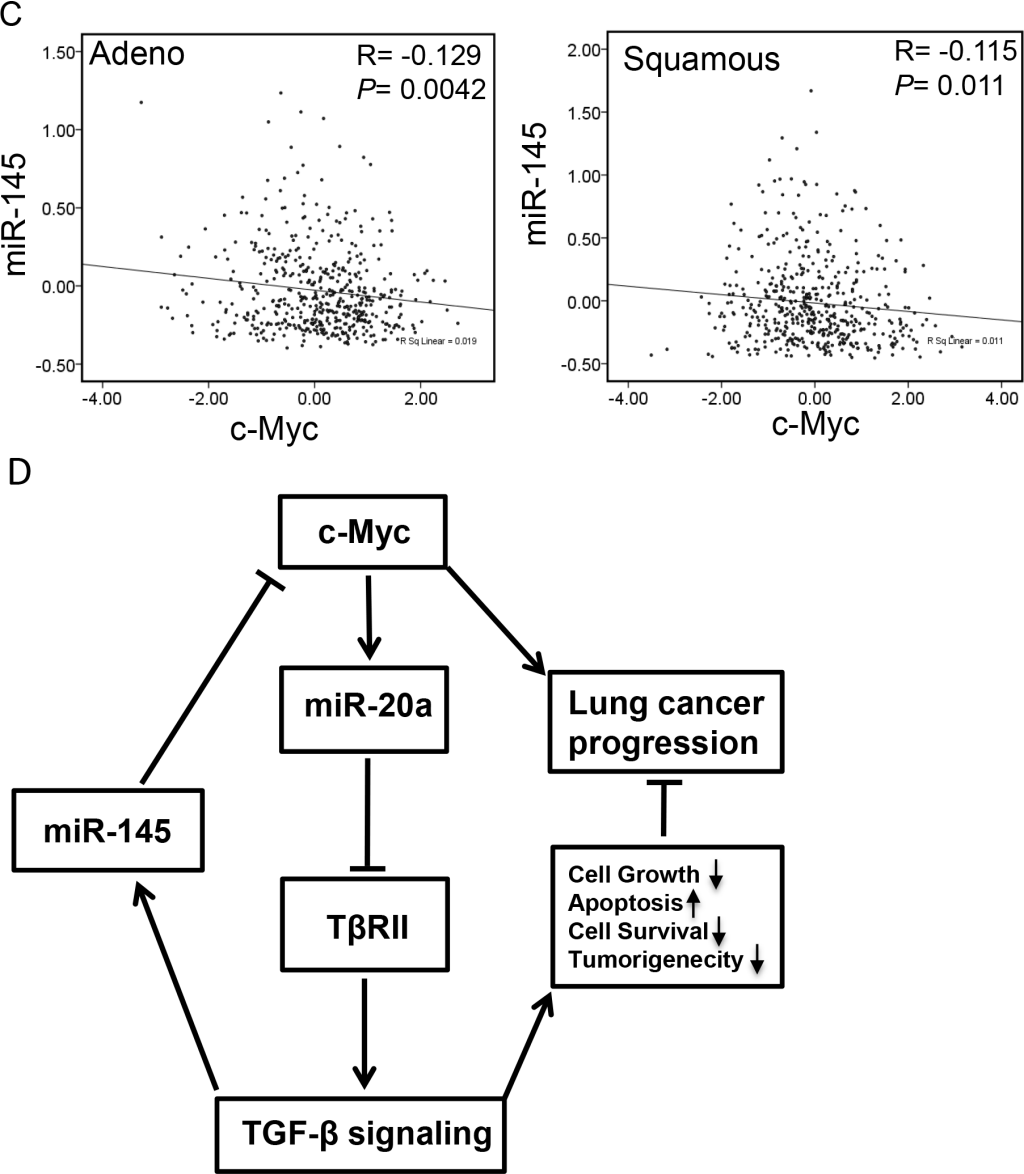
Correlation between the expressions of TβRII and miR-145 and miR-20a and c-Myc in NSCLC **A, B.** and **C.** Data for the expression levels of TβRII, miR-20a, c-Myc and miR-145 from 488 lung adenocarcinoma patients and 490 lung squamous carcinoma patients (TCGA) were analyzed for association by Spearman-rank. A *P*-value of *P* < 0.05 was considered significant. The correlation coefficients (R) and associated *P*-values are shown. The scatter plot demonstrates the correlation between two continuous variables. **D.** Schematic for interaction between c-Myc/miR-20a and TGF-β/miR-145 signaling in lung cancer. Oncogene c-Myc inhibits TGF-β signaling through upregulating miR-20a and decreasing TβRII expression. Inhibition of TGF-β signaling leads to a decreased level of miR-145 and to the loss of TGF-β-induced tumor suppressor functions during lung cancer progression. Downregulation of miR-145 contributes to increased expression of c-Myc resulting in the downregulation of miR-20a.

To verify whether possible associations in the expressions of TβRII, miR-20a, c-Myc and miR-145 are present in lung cancer, we have used the expression data for TβRII, miR-20a, c-Myc and miR-145 from a total of 488 primary lung adenocarcinoma specimens and 490 primary lung squamous carcinoma specimens as described in “Figure 1C”. As shown in Fig. 8, there is a positive correlation between the expression of TβRII and miR-145 (Fig. 8A) in both adenocarcinoma and squamous cell carcinoma. Similarly, we have observed direct correlation between the expressions of miR-20a and c-Myc (Fig. 8B) and inverse correlation between c-Myc and miR-145 expressions (Fig. 8C). These data further support our *in vitro* findings that an auto-feedback loop TGF-β/miR-145/c-Myc/miR-20a/TβRII may be involved in the loss of TβRII expression and TGF-β-induced tumor suppressor functions in lung cancer (Fig. 8D).

## DISCUSSION

The resistance to TGF-β-mediated tumor suppressor functions could be caused in multiple ways involving both genetic and epigenetic changes in TGF-β signaling molecules. While mutations and deletions within coding sequence of TβRII gene are prevalent in colorectal cancers, these are rare in NSCLC. Mutations in Smad2 and Smad4 genes have been found in 5–10% of lung cancers [6, 7]. Gene expression profiles are dependent on changes in the epigenome, including DNA methylation, histone modifications, and noncoding RNA regulation. Many studies have shown that inhibition of DNA methylation, histone deacetylation, and some miRNAs can increase some tumor suppressor gene expression in lung cancer [9, 10, 29]. The loss of expression of TβRII protein is thought to be one of the main reasons in the impairment of TGF-β-induced tumor suppressor functions in lung cancer. This is supported by our previous report that stable expression of TβRII in TGF-β unresponsive cells restores TGF-β-induced inhibition of cell proliferation, induction in apoptosis, and decrease in tumorigenicity [8]. We have also shown that there is a high level of histone deacetylation in the promoter of TβRII gene and histone deacetylation inhibitors can, in part, restore the expression of TβRII in lung cancer cells [11]. Unlike the underlying genome that is largly unchanged in normal conditions within an individual, the epigenome can be dynamically altered by environmental conditions, genetic background, and signaling crosstalk. In an attempt to investigate what other mechanisms are involved in the downregulation of TβRII in lung cancer, we found that miR-20a, a miRNA encoded by the miRNA-17–92 cluster, downregulates TβRII expression, suggesting that multiple mechanisms are associated with TβRII regulation in lung cancer. In this study, the salient features are (1) to our knowledge, this is the first study, to determine the role of miR-20a in suppressing antitumor effects of TGF-Δ in NSCLC through downregulation of TβRII by binding to its 3′-UTR; (2) to study the mechanism of how c-Myc is involved in the regulation of TβRII through the regulation of miR-20a expression; and (3) to investigate how the loss of miR-145 in lung cancer cells can demote TGF-β signaling through regulating miR-20a and TβRII expression.

Differential expression of miRNAs in cancers plays an important role in tumor development and progression by regulating tumor suppressor genes and oncogenes. Our miRNA microarray analyses and northern blot analyses indicate that miR-20a expression is significantly higher in lung cancer cell lines lacking TβRII in comparison to lung cancer and epithelial cell lines with normal TβRII expression. This is supported by the observation that there is an inverse correlation between miR-20a and TβRII expression in lung tumors (Fig. 1). This is in agreement with the previous report suggesting an upregulation of miR-20a in lung cancer [30]. This specific effect of miR-20a on TβRII regulation was further determined by its overexpression in lung cell lines with TβRII expression and by its underexpression in cell lines without TβRII expression (Fig. 2 and 5). It is well-known that TβRII is central to TGF-β signaling, and it is required for the antitumor effects of TGF-β. Moreover, we have shown that stable expression of TβRII in TGF-β-unresponsive cells restores TGF-β-induced inhibition of cell proliferation, induction in apoptosis, and decrease in tumorigenicity [8]. Interestingly, overexpression of miR-20a in three lung epithelial cell lines with normal TβRII levels resulted in the inhibition of tumor suppressor functions of TGF-β through downregulation of TβRII expression (Fig. 4). Conversely, inhibiting miR-20a in two lung cancer cell lines lacking TβRII decreases tumorigenicity of cells through restoring TβRII expression and TGF-signaling (Fig. 5 and S3). The specificity of these effects of miR-20a through TβRII/TGF-β signaling was further supported by the fact that the reduction in tumorigenicity by knocking down miR-20a was attenuated by TGF-β receptor kinase inhibitors (Fig. S4). These findings suggest that lung cancer cells could escape from the autocrine antitumor effects of TGF-β and that Smad signaling is intact in these cell lines. Previous studies show that miR-20a inhibits apoptosis by targeting E2F1 and Fas [31, 32]. According to our findings, miR-20a inhibits tumor cell apoptosis and promotes tumor cell growth and tumorigenicity in part through downregulating TβRII and attenuating TGF-β signaling.

A tumor could be caused by the imbalance between oncogenic and tumor suppression functions of different genes. Acquired resistance to TGF-β-induced antitumor effects is a key step in the early stages of tumorigenesis [3]. Some oncogenic pathways can attenuate TGF-β mediated tumor suppressor function, such as Notch1 and c-Myc [33, 34]. Our study shows oncogenic c-Myc inhibits TβRII expression by upregulating miR-20a, resulting in a loss of TGF-β-mediated tumor suppressor functions in lung cancer (Fig. 6). Upregulation of c-Myc in NSCLC [35] reinforces the notion that it may play a role in the development of NSCLC through inactivation of TGF-Δ signaling. This is supported by the fact that c-Myc activation inhibits TGF-β signaling through miR-17~92 microRNA cluster in colorectal cancer [22].

Recent studies show that interaction between TGF-β/Smad signaling and miRNAs is involved in the development of diseases, such as cardiovascular diseases, pulmonary diseases, and cancer [36–38]. TGF-β/Smad signaling can regulate expression of many miRNAs through transcriptional level or posttranscriptional level [39]. Some miRNAs also affect TGF-β signaling activity by regulating expression of some important components in TGF-β/Smad signaling pathway [34, 37]. Our current study shows that TGF-β promotes miR-145 expression in lung epithelial cells with TΔRII expression. MiR-145 functions as a tumor suppressor, and its expression is decreased in various types of cancers including lung cancer [28, 40]. We hypothesized that TGF-β signaling inactivation may be responsible for decreased expression of miR-145 in lung tumors. We have observed that there are higher levels of miR-145 and lower levels of c-Myc in the immortalized lung epithelial cell lines HPL1A and Beas2B and the lung cancer cell line A549 with normal expression of TβRII compared with those in lung cancer cell lines ACC-LC-176 and VMRC-LCD lacking TβRII (Fig. 7 and S7). This is in agreement with a previous report showing that oncogenic c-Myc is a target of miR-145 in NSCLC cell lines [26]. Overexpression of miR-145 inhibits c-Myc expression in ACC-LC-176 and VMRC-LCD cells leading to upregulation of TβRII and activation of TGF-β signaling. As shown in Fig. 8D, c-Myc activation inhibits TβRII expression by upregulating miR-20a leading to inactivation of TGF-β signaling and miR-145 downregulation. MiR-145 downregulation further promotes upregulation of c-Myc. In agreement with these data, we have observed significant direct correlation between the expression of miR-145 and TΔRII, and c-Myc and miR-20, and inverse correlation between miR-145 and c-Myc, and miR-20a and TΔRII in both adenocarcinoma and squamous cell carcinoma using TCGA database (Fig. 1 and Fig. 8). Such a regulatory model of an auto-feedback loop between c-Myc/miR-20a and TGF-β/miR-145 signaling pathways in regulating TβRII expression may contribute to the development of TGF-β resistance in lung cancer. Besides lung cancer, high levels of miR-20a and c-Myc and decreased expression of TβRII and miR-145 are also found in other types of cancers, such as colorectal cancer, breast cancer [41–47]. Therefore, interaction between c-Myc/miR-20a and TGF-β/miR-145 might also play a role in the downregulation of TβRII in those cancers.

In summary, we have identified, using microRNA microarray, miR-20a as a regulator of TβRII expression and of TGF-β-iduced tumor suppressor functions in NSCLC. This study provides a mechanism of the regulation of c-Myc by miR-145 induced by TGF-β, which in turn downregulates miR-20a. These results demonstrate, for the first time, a mechanism for the loss of TβRII expression in NSCLC through a complex auto-feedback loop TGF-β/miR-145/c-Myc/miR-20a/TβRII. This understanding of multiplex network of oncogenic and tumor suppressor miRNAs and cellular proteins in sending converging signals to tumors may be helpful in designing strategies for molecular targeted therapy based on miRNAs.

## MATERIALS AND METHODS

### Cell lines

Human lung cancer cell lines A549, NCI-H460, VMRC-LCD and ACC-LC-176 and immortalized human lung epithelial cell lines Beas2B and HPL1A were maintained in RPMI 1640 with 10% FBS. To establish stable cell lines with miR-20a overexpression or miR-20a inhibitor, A549, Beas2B and HPL1A cells were infected with lenti-virus expressing miR-20a or GFP lenti-virus and ACC-LC-176 and VMRC-LCD cells with lenti-virus expressing miR-20a inhibitor or control lenti-virus, respectively. 48 h after infection, cells were selected by 1–2 μg/ml puromycin for 10–14 days. Positive clones were tested and maintained in RPMI 1640 medium with 0.5–1 μg/ml puromycin.

### Reagents and antibodies

TGF-β1 was purchased from R&D Systems (Minneapolis, MN). Human has-miR-20a mimics and control miRNA mimics were from Thermo Scientific Dharmacon (Lafayette, CO). Antibodies were purchased as follows: anti-Smad2, anti-Smad3, anti-phospho-Smad2 and anti-phospho-Smad3 from Cell Signaling (Denver, MA); anti-p21^CIP1^, anti-Smad4, anti-c-Myc and anti-TβRII from Santa Cruz Biotechnology (Santa Cruz, CA); anti-Ki67 from VECTOR (Burlingame, CA); and anti-β-actin from Sigma Biochemicals (St Louis, MO). The TRKI (LY2109761) was kindly provided by Dr. Jonathan Yingling (Eli Lilly Pharmaceuticals, Indianapolis, IN).

### Immunoblot analysis

Western blotting was performed as previously described [48].

### Plasmids

Has-miR-20a lentivector and the inhibitor has-miR-20a lentivector were purchased from Applied Biological Materials Inc (Richmond, Canada). Human pri-mir-145 cDNA was amplified by PCR and then inserted into pLenti-III-mir cloning vector through EcoRI and Xhol restriction enzyme sites, leading to establishment of has-miR-145 lentivector. PGL miRNA luciferase reporter vector was constructed by inserting TβRII mRNA 3′ untranslated region (3′UTR) containing miR-20a binding site into pGL3 control vector downstrem of luciferase mRNA (Xba I site). PCGN-c-Myc vector was established by inserting human c-Myc cDNA into pCGN vector. The fragments of the upstream of miR-20a gene promoter were amplified by PCR and then inserted into pGL3 basic vector to construct pGL3-miR-20a promoter 1 vector containing Myc binding site and pGL3-miR-20a promoter 2 vector without Myc binding site.

### Northern blot analysis

Total RNA (20 μg) was resolved on a 12% denatured polyacrylamide gel containing 8M urea. The RNA was then transferred to a Hybond-N+ nylon membrane. After UV cross-linking, the membrane was incubated in prehybridization buffer (200 mM Na_2_HPO_4_ and 7% SDS) at 37°C for more than 30 min and then hybridized with specific α[P^32^]-labeled human miR-20a probe generated using StarFire Nucleic Acid Labeling System (IDT, CA) at 37°C for 24 h. The membrane was washed for 10 min three times with buffer (0.1% SDS, 2xSSPE) and exposed to film. After hybridization with miR-20a probe, the membrane was stripped and reblotted with specific α[P^32^]-labeled human U6 probe (IDT) as a loading controls.

### Quantitative real-time PCR analysis

Taqman probes for RUN48, miR-20a and miR-145 were purchased from Applied Biosystems (Foster City, CA, USA). mRNA levels of p21^CIP1^, PAI-1, TβRII, c-Myc and GAPDH were determined by real-time PCR using SYBR Green master mix kit (Roche, Indianapolis, IN). Primer sequences were as follows: human p21^CIP1^: sense, 5′-G A C A C C A C T G G A G G G T G A C T-3′, antisense, 5′-C A G G T C C A C A T G G T C T T C C T-3′; human TβRII: sense, 5′-G G G G A A A C A A T A C T G G C T G A-3′, antisense, 5′-G A G C T C T T G A G G T C C C T G T G-3′; human PAI-1: sense, 5′-GACATCCTGGAACTGCCCTA-3′, antisense, 5′-GGTCATGTTGCCTTTCCAGT-3; human c-Myc: sense, 5′-C C T A C C C T C T C A A C G A C A G C-3′, antisense, 5′-C T C T G A C C T T T T G C C A G G A G-3′; and human GAPDH: sense, 5′-C G A G A T C C C T C C A A A A T C A A-3′, antisense, 5′-T G T G G T C A T G A G T C C T T C C A-3′.

### Immunohistochemistry

Sections of xenograft tumor tissue were prepared, deparaffinized with xylene, and then rehydrated in water. Antigen retrieval was performed in Tris-EDTA solution, pH 9.0. After incubation in Tris-buffered saline with Tween-20 for 10 minutes and 3% H_2_O_2_ for 10 minutes, the sections were blocked in PBS buffer with 10% goat serum for 2 h. The sections were then incubated with mouse anti-p21^CIP1^, rabbit anti-p-Smad2, rabbit anti-cleaved caspase 3, or rabbit anti-Ki67 antibody overnight at 4°C, secondary antibody with horseradish peroxidase for 1 h and diaminobenzidine (DAB) chromogen, sequentially. Finally, the sections were counterstained with hematoxylin.

### Luciferase reporter assay

Cells were transiently transfected with various constructs and β-Gal vector as indicated using Lipofectamin 2000 (Invitrogen). In each experiment, equal amounts of total DNA were transfected. 48 h after transfection, cells were harvested for luciferase activity assay. Luciferase activity was normalized to β-galactosidase activity and the relative luciferase activity was presented.

### Animal studies

Eight-week-old female nude mice (NU/J, Jackson Labs, Taconic) were inoculated in their right flanks subcutaneously with 4 × 10^6^ control ACC-LC-176 cells or different clones of ACC-LC-176 cells stably transfected with miR-20a inhibitor. Average tumor burden was calculated with calipers in millimeters as the mean tumor diameter measured in two dimensions. Tumor volume was calculated as previously reported [48]. All animal protocols were approved by IACUC.

### Statistical analysis

Results were statistically compared using an unpaired Student's *t*-test or One-Way ANOVA. A *P*-value of *P* < 0.05 was considered significant. Spearman's rank correlation coefficients and corresponding *P* values were used to evaluate association between two continuous variables.

## SUPPLEMENTARY FIGURES


