# Supplementary material for: An epigenetic auto-feedback loop regulates TGF-β type II receptor expression and function in NSCLC
Source: Oncotarget. 2015 Aug 12;6(32):33237–52. doi: 10.18632/oncotarget.4893 (PMC4741762; doi:10.18632/oncotarget.4893)
Supplement: Supplementary file 1 [file oncotarget-06-33237-s001.pdf]

SUPPLEMENTARY FIGURES

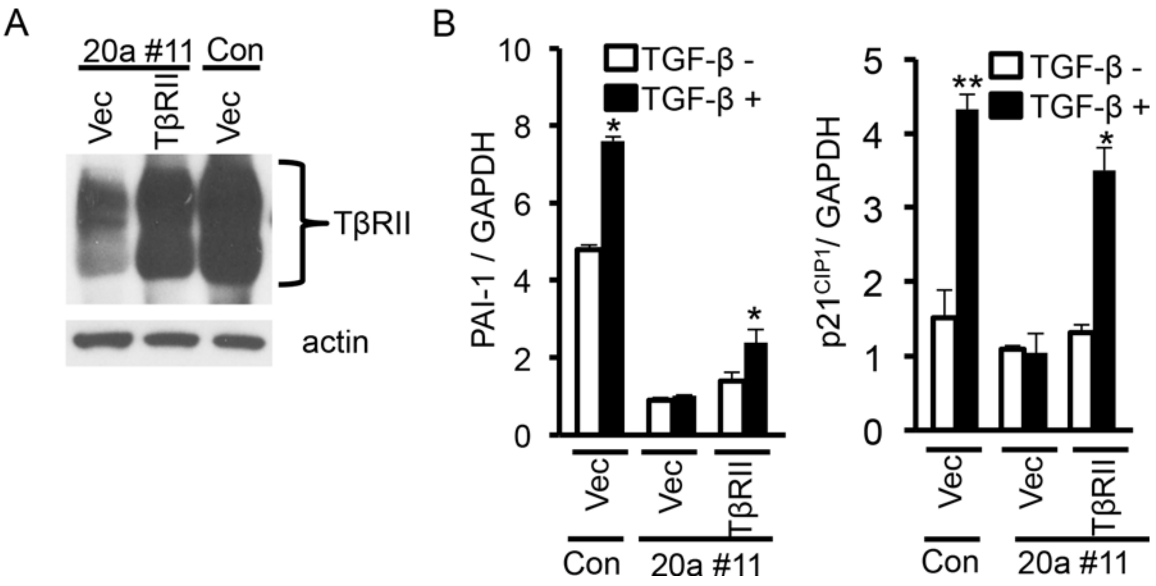

Supplementary Figure S1: TβRII re-expression decreases inhibitory effect of miR-20a on TGF-β signaling target gene expression. A and **B.** Beas2B cells with miR-20a stable expression and control vector cells were transfected with TβRII expression vector or control empty vector. 48 h after transfection, cells were exposed to 5 ng/ml TGF-β for 3 h. TβRII protein expression was analyzed by Western blot analysis (A) P21^{CIP1} and PAI-1 mRNA levels were measured by QRT-PCR. *$P < 0.05$, **$P < 0.01$, *vs*. TGF-β untreated cells in each group (B).

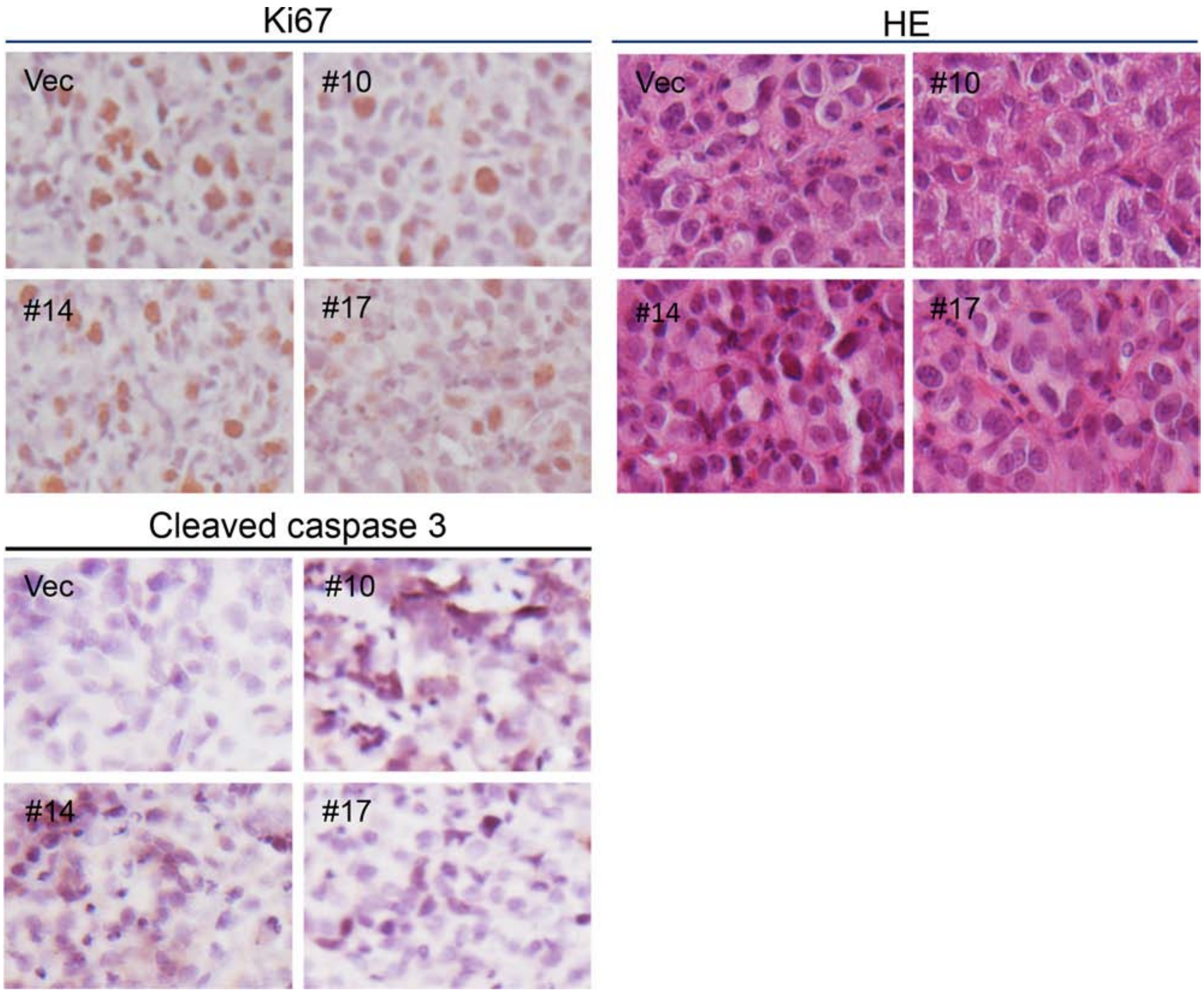

Supplementary Figure S2: Histological analyses of xenograft tumor tissues. Sections from xenograft tumor tissue derived from ACC-LC-176 cells with miR-20a inhibitor or control vector were deparaffinized, and stained with Hematoxylin and Eosin (HE). Immunohistochemical staining for the expression of Ki67 and cleaved Caspase 3 was performed. Representative pictures are shown (X 400).

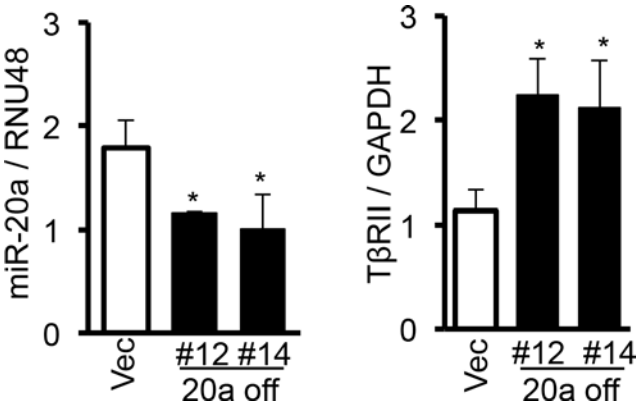

Supplementary Figure S3: Stable knock down of miR-20a upregulates TβRII expression in VMRC-LCD lung cancer cells. Total RNA was purified from VMRC-LCD cells stably expressing miR-20a inhibitor and control vector. MiR-20a and TβRII mRNA levels were analyzed by QRT-PCR ($n = 3$, the mean expression and SD are displayed). *$P < 0.05$, vs. vector (Vec) group.

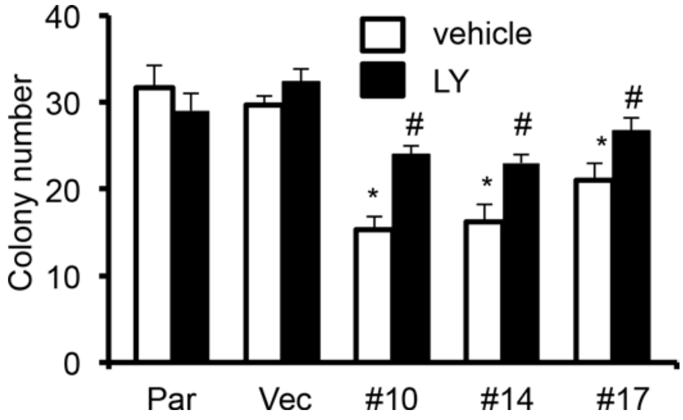

Supplementary Figure S4: Loss of miR-20a function inhibits anchorage-independent growth in lung cancer cell line ACC-LC-176. 4000 cells from #10, #14 and #17 stable clones with miR-20a inhibitor and parental and vector controls (as in Fig. 5) were plated on soft agar. 10 µM LY2109761 (LY) was added on the top agar layer every third day. After 14 days, colonies were counted. Each data point represents the number of colonies from an average of three values. *P < 0.05, *vs*. LY untreated parental (Par) and control vector cells (Vec); #P < 0.05, *vs*. LY untreated cells in each group.

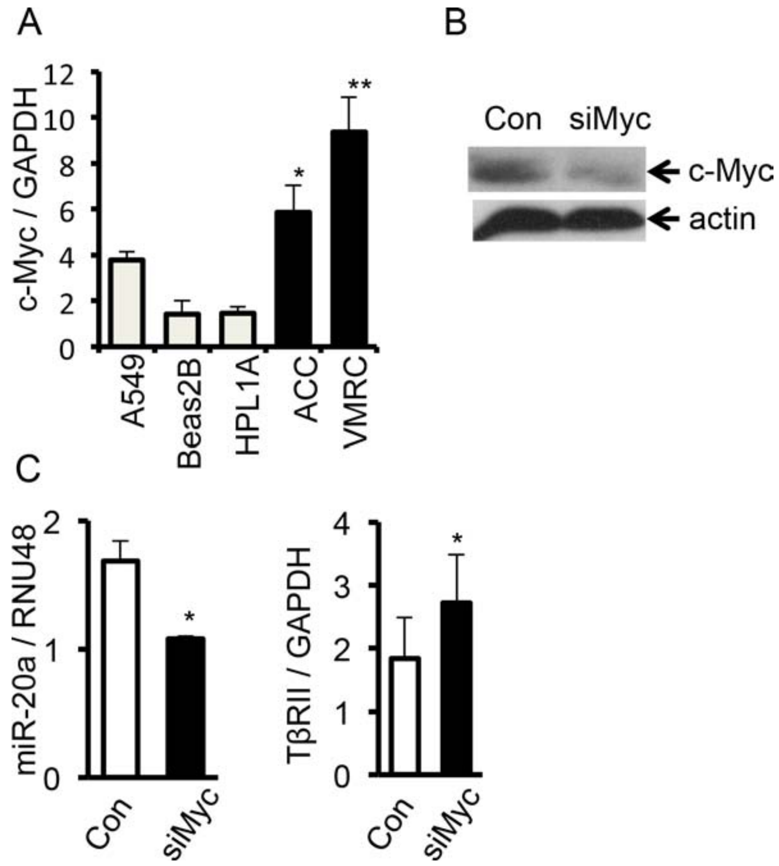

Supplementary Figure S5: C-Myc downregulation inhibits miR-20a expression and increases TβRII level in lung cancer cells. A. Total RNA was purified from A549, Beas2B, HPL1A, ACC-LC-176 (ACC) and VMRC-LCD (VMRC). C-Myc mRNA expression was analyzed by QRT-PCR and normalized by GAPDH ($n = 3$, the mean expression and SD are displayed). $*P < 0.05$, $**P < 0.01$, *vs*. A549, Beas2B and HPL1A cells. **B.** ACC-LC-176 cells were transfected by c-Myc siRNA and control siRNA. 48 h after transfection, cells were harvested for western blotting. β-actin was used as loading control. **C.** Total RNA was purified from cells transfected as in "B". MiR-20a and TβRII expression was analyzed by QRT-PCR and normalized by RNU48 and GAPDH, respectively. $*P < 0.05$, *vs*. control group (Con).

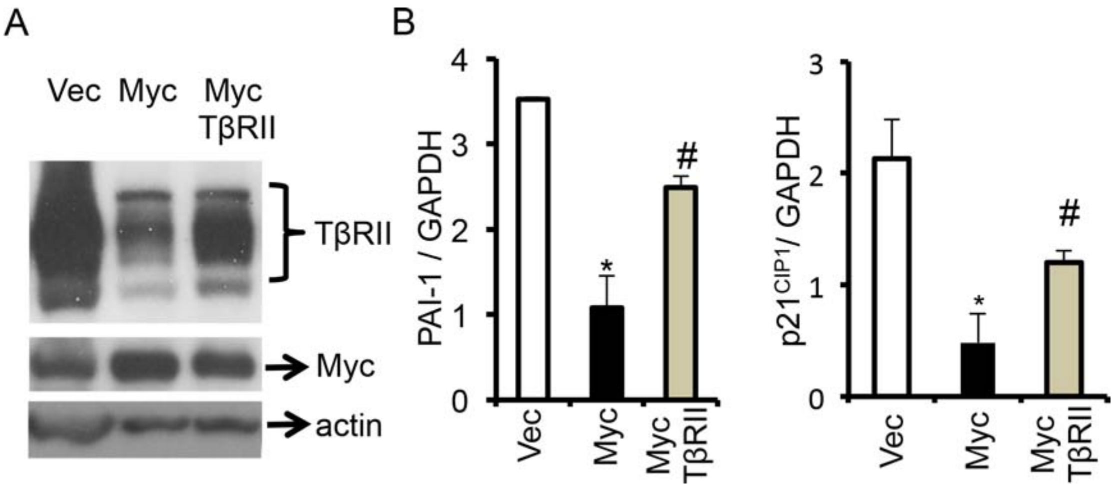

Supplementary Figure S6: TβRII re-expression attenuates inhibitory effect of c-Myc on TGF-β signaling target gene expression. **A** and **B.** HPL1A cells were co-transfected with c-Myc and TβRII expression vectors as indicated. 48 h after transfection, cells were harvested. C-Myc and TβRII protein levels were analyzed by Western blot analysis (A) $P21^{CIP1}$ and PAI-1 mRNA levels were measured by QRT-PCR analyses (B) **P* < 0.05, *vs*. cells transfected with control vector (Vec); #*P* < 0.05, *vs* cells transfected with c-Myc expression vector alone (Myc).

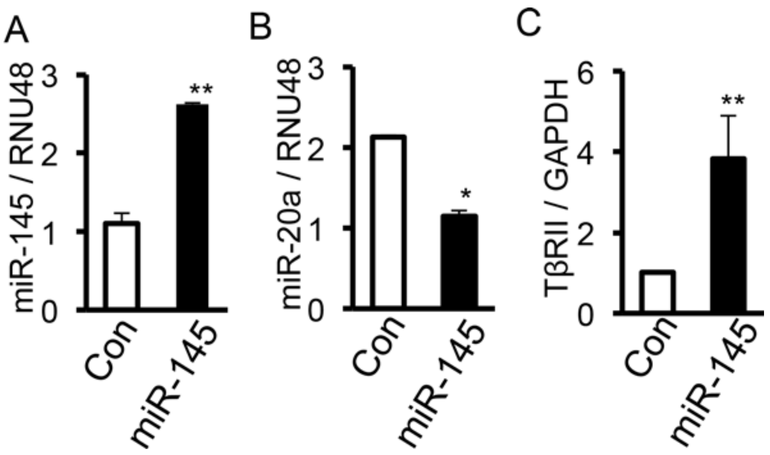

Supplementary Figure S7: Expression of miR-145 restores TβRII expression in ACC-LC-176 cells by downregulating miR-20a. Total RNA was purified from ACC-LC-176 human lung cancer cells stably expressing miR-145 and control vector cells. The levels of miR-145 **A.** miR-20a **B.** and TβRII mRNA **C.** were measured by QRT-PCR and normalized by internal control RNU48 and GAPDH, respectively. *$P < 0.05$, **$P < 0.01$, *vs*. control vector cells (Con).
